# Hospital Epidemiology of Methicillin-Resistant* Staphylococcus aureus* in a Tertiary Care Hospital in Moshi, Tanzania, as Determined by Whole Genome Sequencing

**DOI:** 10.1155/2018/2087693

**Published:** 2018-01-02

**Authors:** Happiness H. Kumburu, Tolbert Sonda, Pimlapas Leekitcharoenphon, Marco van Zwetselaar, Oksana Lukjancenko, Michael Alifrangis, Ole Lund, Blandina T. Mmbaga, Gibson Kibiki, Frank M. Aarestrup

**Affiliations:** ^1^Kilimanjaro Clinical Research Institute, Moshi, Tanzania; ^2^Kilimanjaro Christian Medical Centre, Moshi, Tanzania; ^3^Kilimanjaro Christian Medical University College, Moshi, Tanzania; ^4^DTU-Food, Technical University of Denmark, Copenhagen, Denmark; ^5^Centre for Medical Parasitology, Department of Immunology and Microbiology, University of Copenhagen, Copenhagen, Denmark; ^6^Center for Biological Sequence Analysis, Department of Bioinformatics, Technical University of Denmark, Copenhagen, Denmark; ^7^East African Health Research Commission, Bujumbura, Burundi

## Abstract

**Objective:**

To determine molecular epidemiology of methicillin-resistant* S. aureus* in Tanzania using whole genome sequencing.

**Methods:**

DNA from 33* Staphylococcus* species was recovered from subcultured archived* Staphylococcus *isolates. Whole genome sequencing was performed on Illumina Miseq using paired-end 2 × 250 bp protocol. Raw sequence data were analyzed using online tools.

**Results:**

Full susceptibility to vancomycin and chloramphenicol was observed. Thirteen isolates (43.3%) resisted cefoxitin and other antimicrobials tested. Multilocus sequence typing revealed 13 different sequence types among the 30* S. aureus* isolates, with ST-8 (*n* = seven, 23%) being the most common. Gene detection in* S*.* aureus* stains were as follows:* mecA*, 10 (33.3%);* pvl*, 5 (16.7%);* tst*, 2 (6.7%). The SNP difference among the six Tanzanian ST-8 MRSA isolates ranged from 24 to 196 SNPs and from 16 to 446 SNPs when using the USA300_FPR3757 or the USA500_2395 as a reference, respectively. The mutation rate was 1.38 × 10^−11^ SNPs/site/year or 1.4 × 10^−6^ SNPs/site/year as estimated by USA300_FPR3757 or the USA500_2395, respectively.

**Conclusion:**

* S. aureus* isolates causing infections in hospitalized patients in Moshi are highly diverse and epidemiologically unrelated. Temporal phylogenetic analysis provided better resolution on transmission and introduction of MRSA and it may be important to include this in future routines.

## 1. Introduction

*Staphylococcus aureus* is one of the most important causes of serious infections in humans worldwide [1]. In particular, methicillin-resistant* S. aureus* (MRSA) isolates have become common causes of infections in hospitals as well as community settings.

For quite some time, MRSA has been a worldwide leading cause of nosocomial infections [2, 3]. From the 1960s to early 1970s, MRSA infections were often confined to hospitals and healthcare facilities. During this time mostly the classical or hospital-associated MRSA (HA-MRSA) accounted for these infections. In the mid-1990s, a surge of MRSA infections was reported in community settings among healthy people who had not been in contact with hospital environments [4]. This change was regarded as a result of introduction of a unique MRSA strain, so-called community associated MRSA (CA-MRSA) [5]. The ability of CA-MRSA to cause frequent outbreaks, with high virulence and a high rate of dissemination to different geographical locations, has played a major role in increasing the emergence and distribution of staphylococcal infections globally [6].

Diagnosis of MRSA isolates has not been easy despite the advancement in medical technology. The cefoxitin disk diffusion method is a reliable test for MRSA; however it needs to be supplemented by several other tests [7]. The emergence of high throughput technologies such as whole genome sequencing (WGS) promises to revolutionize today's clinical microbiology practices [8], increasing insight into pathogens and hence coming up with correct diagnosis, treatment, and control measures for disease-causing pathogens [9]. Recently, bench-top WGS has become available and proven to be a very valuable tool in elucidating the epidemiology of bacterial species including MRSA [10–12]. However, the technology is currently still out of reach for clinical laboratories in developing countries [13] and deployed rarely in research setups in these countries [14]. Recently, this technology was introduced at the Kilimanjaro Christian Medical Centre (KCMC) in Moshi, Tanzania, and already during the first test run, nosocomial transmission of* Enterococcus faecalis* was identified [15].

The prevalence of MRSA infections in most African countries seems to be relatively low [2]. However, given the low research intensity on MRSA infections in Africa this data should not be neglected [16].* S. aureus* and MRSA infections contribute to significant health challenges in Tanzania as well [17–19] despite the fact that the reports are few and hence the current prevalence may not be indicating the actual disease burden. It is therefore important to be able to explain the molecular epidemiology of the clones, which are locally circulating. This will contribute to the knowledge on infection transmission, and hence introduction of proper treatment and control programs. Here we report on molecular epidemiology of* S. aureus* in Tanzania using WGS tools located at KCMC. WGS was used to determine sequence types, phylogenetic relation, and comparison to genome sequences that were obtained from ENA (https://www.ebi.ac.uk/ena) and NCBI. Additionally, antimicrobial resistance genes as well as virulence factors were determined.

## 2. Material and Methods

### 2.1. Study Setting, Participants, and Sample Collection

The current study is based on the previously conducted project [20] at Kilimanjaro Christian Medical Centre (KCMC) tertiary care hospital in North-Eastern Tanzania, from August 2013 to August 2015. The study aimed to determine the pattern of bacterial pathogens associated with different health conditions among patients who were admitted to the medical and surgical departments of KCMC. For the study, informed consent was obtained from 575 patients and clinical samples were collected from these patients who were admitted to medical and surgical wards at KCMC during the period defined above. Demographic data of these patients were obtained from their patient hospital files. Relevant information was captured on designated case record forms (CRFs) and double entered in OpenClinica (OpenClinica LLC and collaborators, Waltham, MA, USA). Data was extracted and exported to STATA 13 (StataCorp LP, Texas 77845, USA) for data analyses.

### 2.2. Ethical Consideration

Ethical approval for the study was obtained from National Institute for Medical Research with Certificate number NIMR|HQ|R.8a|Vol.IX/2080 and from Kilimanjaro Christian Medical University College with Certificate RECC number 891.

### 2.3. Laboratory Methods and Data Analysis

In the previous study, the clinical samples collected from the 575 patients during routine clinical care were transported to the microbiology unit of the Kilimanjaro Clinical Research Institute (KCRI) Biotechnology Laboratory for routine classical microbiology processes. Both Gram negative and positive bacteria were observed.* S. aureus* was the most predominant among Gram positive isolates. Antimicrobial susceptibility testing was performed using disk diffusion techniques as described in the previous study [20]. All isolates from the previous study were stored at −80°C for whole genome sequencing (WGS). For the current study, we recovered 30 out of 35* S. aureus* isolates upon subculturing. All 30 isolates were subjected to molecular biology lab for DNA isolation. We also retrieved coagulase negative staphylococci (CoNS) isolates from the archive. However, only CoNS of clinical importance were included in this study [21, 22]. Genomic DNA was extracted using MasterPure™ Gram Positive DNA Purification Kit Cat. No. MGP04100, Epicentre, Illumina. The quality and quantity of genomic DNA were confirmed using Qubit 2.0 fluorometer, (Thermal Fisher Scientific, Waltham, MA, USA). Library preparation (dual indexing) was done using NexteraXT DNA Preparation Kit (Illumina Inc., San Diego, CA, USA).

Whole genome sequencing of the library was completed on Illumina Miseq using a paired-end 2 × 250 bp protocol.

### 2.4. Multilocus Sequence Typing (MLST), Antimicrobial Resistance, and Virulence Genes

The raw reads were de novo assembled through the assembly pipeline (version 1.0) available from the Center for Genomic Epidemiology (CGE) (https://cge.cbs.dtu.dk/services/) which is based on the Velvet algorithms for de novo short reads assembly [23]. The assembled sequences were analyzed using bioinformatics tools to identify MLST sequence type (MLST version 1.7) [24], acquired antimicrobial resistance genes (ResFinder version 2.1) [25], and virulence genes (VirulenceFinder version 1.0) [26]. All tools were available online at https://cge.cbs.dtu.dk/services and https://www.ebi.ac.uk/ena.

### 2.5. Single Nucleotide Polymorphisms (SNPs) and Temporal Bayesian Phylogenetic Trees

Phylogenetic analyses were performed on 40 ST-8* S*.* aureus* genomes. Six ST-8* S. aureus* genomes were from the current study. These were found to harbor the* mecA *gene (hence MRSA) and it was therefore important to gain better understanding of their genetic relation. For global comparison, 34 ST-8 MRSA genomes were retrieved from ENA and NCBI. These 34 genomes were from five different studies [27–30]. A much larger number of genomes have been deposited, but we restricted the set to only include genomes for which epidemiological information was available. Raw sequence data of the 6 ST-8* S*.* aureus* from our study has been submitted to the European Nucleotide Archive (https://www.ebi.ac.uk/ena) under study accession number PRJEB23314. Information pertaining to all 40 ST-8 MRSA used for construction of phylogenetic trees in this study is reported in the supplementary information (SI) appendix, Tables 1a and 1b.

#### 2.5.1. Single Nucleotide Polymorphisms

SNPs were identified using the CSI phylogeny [31, 32] pipeline, available on CGE (http://www.genomicepidemiology.org). The raw reads were mapped to two reference genomes,* S. aureus* USA300_FPR3757 (accession number CP000255, chromosome length 2,917,469 bp) and USA500_2395 (accession number CP007499, chromosomal length 2,955,646) using BWA version 0.7.2 [33]. The mpileup tool from SAMTools version 0.1.18 [34] was applied to determine SNPs. SNPs were filtered out when not matching the following criteria: (1) a minimum distance of 10 bp between SNPs, (2) a minimum of 10% of the read depth at SNP positions, (3) mapping quality above 25, and (4) SNP quality above 30. All indels were excluded. The SNPs from each genome were concatenated to single alignment corresponding to position on the reference genome. The concatenated sequences were used for constructing a maximum likelihood tree using FastTree [35].

#### 2.5.2. Temporal Bayesian Phylogenetic Tree

Prior to reconstructing a phylogenetic tree in BEAST, concatenated SNPs were examined for significant recombination sites. We used a novel hidden Markov model tool, RecHMM [36], to detect clusters of sequence diversity that mark recombination events within branches. SNPs in these regions were excluded in the temporal phylogenetic analysis. SNP sequences were then used for reconstructing a temporal phylogenetic tree using Bayesian Evolutionary Analysis Sampling Trees (BEAST) version 1.8.3 [37] to estimate mutation rate and divergence time. Test models with combinations of different population size change and molecular clock were evaluated to identify the best-fit model. This was achieved by performing different BEAST runs with different selected models. Comparison of the log files from all models in the output runs was done in Tracer, a program that compares all BEAST runs and gives a score (ACT score). The temporal tree with the highest posterior probability was then constructed using the best-fit model (random local clock and coalescent Bayesian skyline). The BEAST MCMC chains were simulated for 300 million steps and subsampled every 10,000 steps. The final maximum clade credibility (MCC) was determined using TreeAnnotator [37] with 10% of the MCMC steps discarded as burn-in. Statistical confidence was represented by the 95% highest posterior density (HPD) interval. Mutation rate and divergence time were estimated by BEAST and the population size over time was estimated using the Bayesian skyline plot implemented in Tracer [37]. The effective population size was inferred by the product of the interval size (*γ*_*i*_) and *i*(*i* − 1)/2, where *i* is the number of genealogical lineages in the interval [38, 39].

## 3. Results

### 3.1. Isolates Characteristics

Thirty* S. aureus* and three coagulase negative staphylococci (CoNS) isolates from patients who were admitted to wards of medical and surgical departments were whole genome sequenced. Of the thirty* S. aureus* isolates 8, 14, and 8 were isolated in 2013, 2014, and 2015, respectively. The three CoNS isolates were all isolated in year 2015. Twenty-nine bacterial isolates were recovered from wound swabs, 3 isolates from blood culture, and 1 from sputum. The causes of the wounds were as diverse as indicated in Table 1.

### 3.2. Phenotypic Antimicrobial Resistance Pattern of* S. aureus* Isolates

All* S. aureus* tested were susceptible to vancomycin and chloramphenicol. Thirteen (43.3%) isolates were resistant to cefoxitin while resistance to trimethoprim-sulfa, erythromycin, and penicillin was observed for 16 (53.3%), 14 (46.7%), and 30 (100%) isolates, respectively, Table 2.

### 3.3. Multilocus Sequence Typing (MLST), Antimicrobial and Virulence Genes

**Figure 1:**
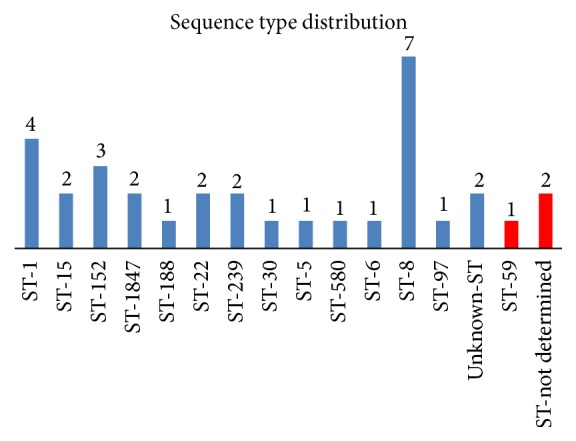
Distribution of sequence types (ST) among* Staphylococcus aureus* and coagulase negative* Staphylococcus* species (CoNS). Fourteen different sequence types were identified, with sequence type 8 being the most common. ST-59 and undetermined ST were observed among the CoNS (presented in red).

In silico multilocus sequence typing revealed 13 different sequence types (ST) among the thirty* S*.* aureus* isolates. There were seven* S*.* aureus* isolates (23.3%) with ST-8, four (13.3%) with ST-1, and three (10%) with ST-152. The remaining isolates had diverse sequence types (see Figure 1).

The three CoNS were one* S. epidermidis* with ST-59 and two* S. haemolyticus* of undetermined sequence type.

Presence of* mecA* was established in 13 (39.4%) of all* Staphylococcus *spp. isolates involved in this study. Ten (33.3%) of the thirty* S. aureus* isolates found to harbor* mecA,* thus classified as MRSA. Six out of the ten* S. aureus* isolates with mecA were ST-8, two were ST-239, and the remaining two had unknown sequence types. All three CoNS possessed the* mecA* gene as well. Additionally, five* S. aureus* isolates (16.7%) were found to harbor Panton-Valentine Leukocidin* (pvl)* virulence genes with absence of* mecA*. Two (6.7%) were found to have* tst* genes for toxic shock syndrome. Other antimicrobial and virulence genes possessed by all isolates are further described in Table 3.

### 3.4. Single Nucleotide Polymorphisms (SNPs)

**Figure 2:**
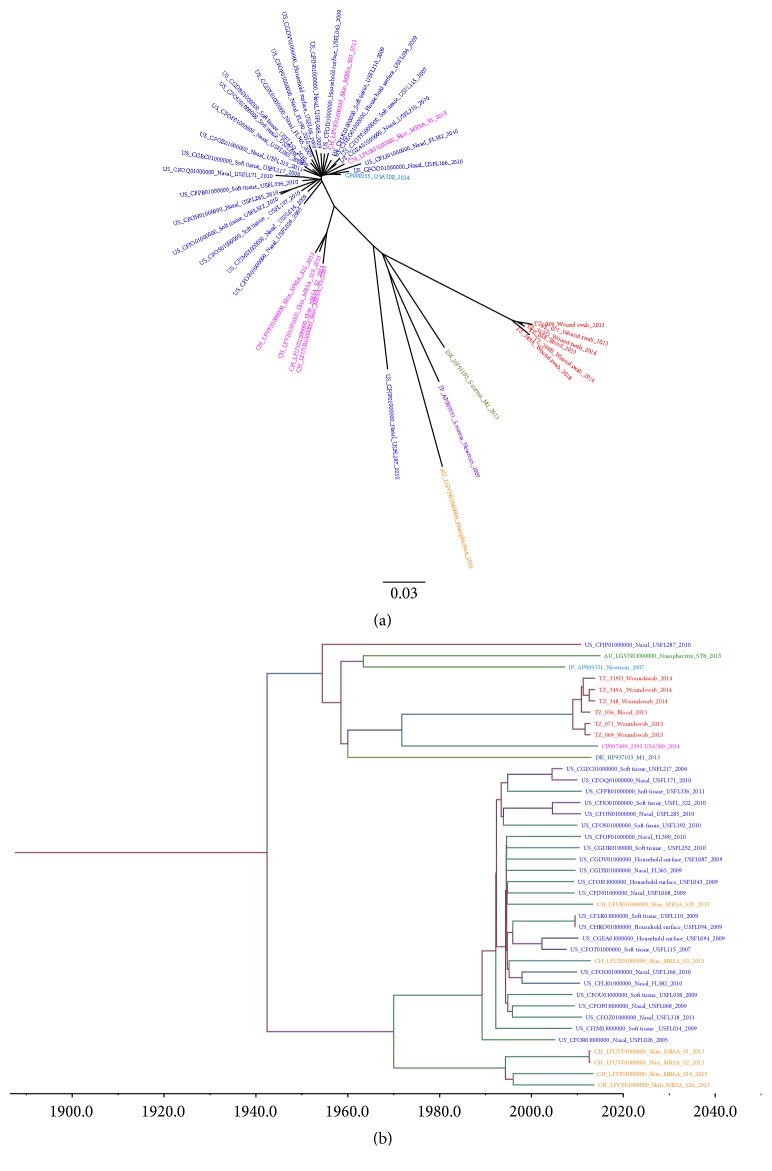
(a)* Maximum likelihood SNP phylogenetic tree of forty ST-8 S*.* aureus genomes*. Raw reads from genomes were mapped to a reference genome of* Staphylococcus aureus* USA300_FPR3757 (accession number CP000255, chromosome length 2,917,469 bp) to generate 3511 qualified SNPs. SNPs generated from each genome were concatenated to single alignment corresponding to position of the reference genome. The concatenated sequences were used for constructing a maximum likelihood tree using FastTree. The tree was visualized by using FigTree version 1.4.0. Of the forty genomes, 6 were from Tanzania (TZ) presented in red; 23 from United States (USA) presented in blue; 6 from Switzerland (CH) presented in pink; one from Japan (JP), Denmark (DK), and Australia (AU) presented in magenta, green, and orange, respectively. USA500_2395 strain genome was included and presented in aqua blue. (b)* Bayesian temporal phylogenetic tree of the 40 MRSA genomes from Tanzania (TZ), United States (USA), Switzerland (CH), Japan (JP), Australia (AU), and Denmark (DK)*. A total of 3148 (90%) SNP sequences were used for reconstructing Bayesian temporal phylogenetic tree using Bayesian Evolutionary Analysis Sampling Trees (BEAST) version 1.8.3. USA300_FPR3757 (accession number CP000255, chromosome length 2,917,469 bp) was used as reference during SNP generation. The mutation rate estimated by BEAST was 1.32 × 10^−11^ SNPs/site/year.

The mapping of the raw reads of these 40 genomes to the reference genome,* S. aureus* USA300_FPR3757, detected 3,511 qualified SNPs. The maximum likelihood SNP tree was constructed using these 3,511 SNPs (Figure 2(a)). RecHMM detected 353 significant recombination SNPs. The remaining SNPs (*n* = 3,148, 90%) were used to construct a temporal phylogenetic tree (Figure 2(b)).

**Figure 3:**
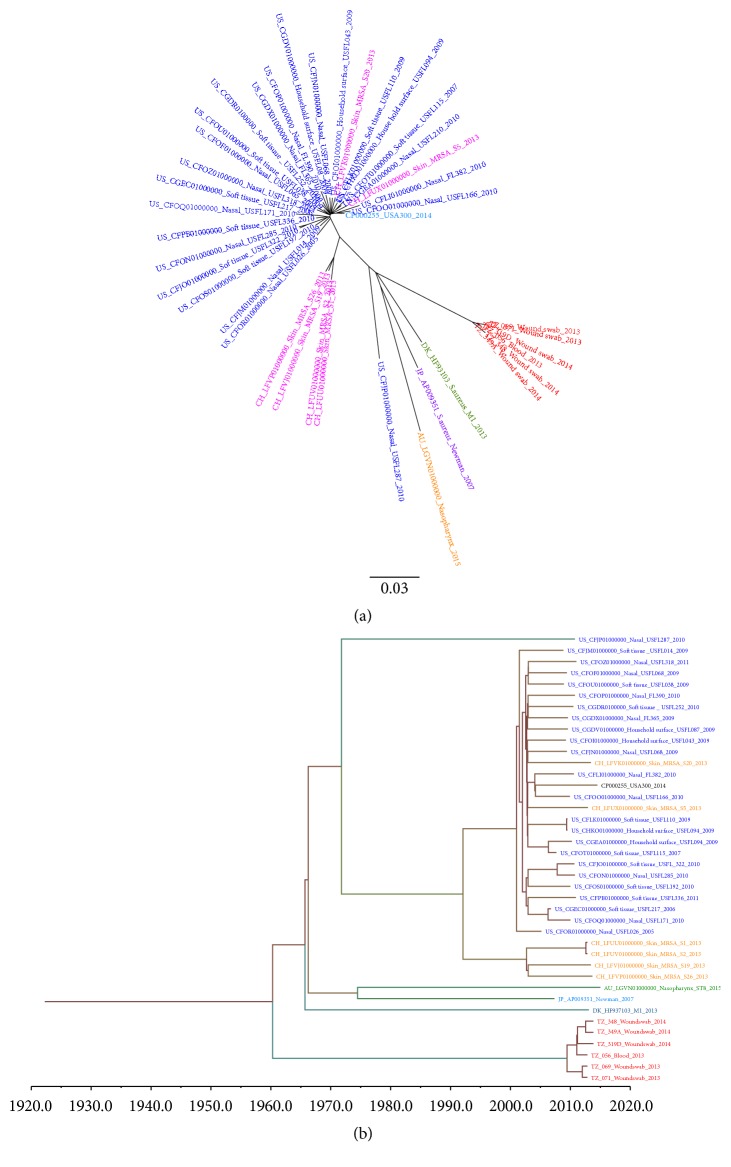
(a)* Maximum likelihood SNP phylogenetic tree of forty ST-8 S*.* aureus genomes*. Raw reads from genomes were mapped to a reference genome of* Staphylococcus aureus* USA500_2395 (accession number CP007499, chromosomal length 2,955,646) to generate 3385 qualified SNPs. SNPs generated from each genome were concatenated to single alignment corresponding to position of the reference genome. The concatenated sequences were used for constructing a maximum likelihood tree using FastTree. The tree was visualized by using FigTree version 1.4.0. Of the forty genomes, 6 were from Tanzania (TZ) presented in red; 23 from United States (USA) presented in blue; 6 from Switzerland (CH) presented in pink; one from Japan (JP), Denmark (DK), and Australia (AU) presented in purple, green, and orange, respectively. USA300_FPR3757 strain genome was included and presented in aqua blue. (b)* Bayesian temporal phylogenetic tree of the 40 MRSA genomes from Tanzania (Red), USA (Blue), Switzerland (CH), Japan (JP), Australia (AU), and Denmark (DK)*. A total of 3029 (89%) SNP sequences were used for reconstructing Bayesian temporal phylogenetic tree using Bayesian Evolutionary Analysis Sampling Trees (BEAST) version 1.8.3.* Staphylococcus aureus* USA500_2395 (accession number CP007499, chromosomal length 2,955,646) was used as reference during SNP generation. The mutation rate estimated by BEAST was 1.4 × 10^−6^ SNPs/site/year.

Mapping the 40 genomes to the second reference genome,* S. aureus* USA500_2395, detected 3,385 qualified SNPs. RecHMM detected 356 significant recombination SNPs. The maximum likelihood SNP tree was constructed using these 3385 SNPs (Figure 3(a)). The remaining SNPs (*n* = 3,029, 89%) were used to construct a temporal phylogenetic tree (Figure 3(b)). From the SNP phylogenetic trees, it seems that the six Tanzanian isolates form their own clade and are closely clustered together. The SNP difference among the six Tanzanian MRSA isolates ranged from 24 to 196 SNPs when using the USA300_FPR3757 as a reference, whereas with the USA500_2395 reference SNP differences ranged between 16 and 446 SNPs. However, the tree topology of the two SNP phylogenetic trees remains basically unchanged. Further information of the SNP differences among all genomes used in construction of the phylogenetic trees is indicated in supplementary material Tables 1a and 1b.

### 3.5. Temporal Bayesian Phylogenetic Trees

The temporal Bayesian phylogenetic tree using USA300_FPR3757 estimated the emergence of the most recent common ancestor to be in ~1942. Primarily the tree is divided into two main clusters in ~1954 (minor) and in ~1969 (major). The minor cluster shows less branching until ~1971 where two other clusters appear, one cluster composing the Tanzanian isolates and the other the USA500 isolate. Further evolution took place to form another lineage in ~2009 when the most recent common ancestor of Tanzanian MRSA isolates seemed to emerge. Subsequent lineages further emerged in ~2010, 2011, and 2012 among Tanzanian MRSA isolates. The 1969 major cluster is more complex compared to the minor cluster. From 1969 further evolution took place in ~1989 and in ~1994. However, from the tree it is predicted that most of the lineages that evolved in the USA and Switzerland emerged around 1990s (Figure 1a in supplementary material).

Using USA500_2395 as the reference, the tree estimates that the most recent common ancestor emerged around ~1960. The tree is also divided into two major clusters in ~1965 (major cluster) and ~2009 (minor). The major cluster is further divided to form subsequent lineages. In ~1992 this major cluster was divided into two other clusters forming in ~2001 and ~2002. The 2001 cluster is mainly composed of USA strains and few from Switzerland, while the 2002 cluster contains only Switzerland strains. The minor (2009) lineage is composed of Tanzanian strains only, which shows introduction of new strains in early and late ~2011 and thereafter further evolution in ~2012 (Figure 1b in supplementary material). However, the infection observed in the hospital in 2013 was due to strains evolved in early and late ~2011 while 2014 infections was due to strains evolved in both 2011 and 2012. Bayesian analysis also shows that the common ancestor between Tanzanian isolates and USA500_2395 emerged around 1971, while with USA300_FPR3757 this emerged around 1961. The mutation rates estimated from the BEAST were 1.38 × 10^−11^ SNPs/site/year and 1.4 × 10^−6^ SNPs/site/year using USA300 and USA500 as references, respectively.

## 4. Discussion

*S. aureus* is an important causative agent of hospital-associated infections worldwide. Studying the pathogen at its molecular level has the potential to bring about breakthroughs in diagnosis, treatment, and infection control. In the current study we determined the molecular epidemiology of* S. aureus *isolates using the next generation whole genome sequencing facility available at KCMC-KCRI Biotechnology Laboratory in Moshi, Tanzania.

Multilocus sequence typing revealed 13 different sequence types among the 30* S. aureus* isolates. This diversity suggests that* S. aureus* infections in the hospitalized patients that were sampled were not epidemiologically related. This is in line with similar findings in other studies in Africa [40] and other parts of the world [41]. The high genetic diversity of* S. aureus* lineages confers their high adaptability to different environments resulting in widespread distribution [42]. Sequence type 8 MRSA which has been observed to be the predominant strain in our hospital is also considered to be the most studied CA-MRSA [43]. The strain has been observed in other parts of the world [44–46] as a cause of infections in both hospital and community settings [43].

Resistance of* S. aureus* to the antimicrobial agent cefoxitin can be used to predict existence of MRSA [47]. This study reports resistance to cefoxitin to be 43.3% by using classical microbiology techniques. The prevalence was further narrowed down to 33.3% when detecting* mecA* using whole genome sequencing. These findings are not far from what has been reported in other studies in Tanzania, as it seems that the prevalence of MRSA in Tanzania is above 10% but less than 50% [17–19, 48–51] in hospitalized patients and ranges between 1.5% and 2.1% in healthy individuals [19, 52]. The prevalence of MRSA infections observed in most African countries ranges between 25% and 50% [2], which is lower than what is seen on other continents [53].

Several virulence genes including* pvl* and* tst *were observed in the isolates studied. Some of these genes may have influenced the severity of the* S. aureus* infections. It then becomes necessary to properly identify the genes for the purpose of controlling and monitoring their spread. In our study we reported five isolates identified to carry* pvl* genes with one of them also possessing the* tst *gene. All five isolates were methicillin susceptible* S. aureus*. Such findings correlate with other studies, which indicate that approximately 50% of MSSA isolates from Africa carry* pvl* genes [54]. However the findings are in contrast with a study conducted in Tanzania whereby ST-88 and ST-1797 were found to be dominant MRSA clones, and* pvl* genes were detected in ST-88 MRSA isolates [50].

Regarding determination of antimicrobial resistance and virulence genes in* S. aureus*, tracing genetic relation of clones circulating in a certain locality is vital key for determining the evolution history [55]. Phylogenetic analysis additionally provides insight into the current and possible future composition of the organism's population. The phylogenetic analysis of ST-8 strains from Tanzania using two different references clearly demonstrated that they belong to their own clade in global epidemiology. The two maximum likelihood SNP trees did not differ significantly in topology. However, the temporal phylogenetic analysis suggests that the USA500_2395 is closer related to Tanzanian ST-8 MRSA than USA300_FPR3757 despite the fact that these two strains are closely related [28]. The temporal phylogenetic analysis based on the USA500-2395 reference suggests that the MRSA infection observed at KCMC hospital was due to multiple introduction events. Three strains emerged at the beginning and end of ~2011; two caused infection in 2013, and one stayed without causing infection in that year. The third together with the one emerged in 2012 caused infections in 2014.

These results illustrate the value of temporal phylogeny in phylogenetic analysis. It provides a framework for examining evolution and molecular diversity in a more comprehensive way, by suggesting approximate times at which divergence occurred. However, the analysis is computationally expensive, taking several days.

## 5. Conclusion

This study showed that* S. aureus* isolates causing infections in hospitalized patients in Moshi, Tanzania, are highly diverse and likely not epidemiologically related. Using temporal phylogenetic analysis provided a higher resolution insight into the transmission and introduction of MRSA, and it may be valuable to include this analysis in future routine analyses despite its computational cost. This study demonstrates the feasibility and value of high throughput technologies in a resource-constrained setting. We propose that technologies such as NGS should be deployed in particular in infectious disease-endemic settings. This will improve diagnosis, treatment, and control of infections in these areas. Just as most of Africa skipped land-line telephony to jump straight to ubiquitous mobile phone use, next generation sequencing technology may provide a similar leapfrog in infectious disease diagnosis, treatment, and control.

## Figures and Tables

**Table 1:** Isolate characteristics.

Sample name	Species	Collection date	Diagnosis	Specimen	Ward	Room number	Bed number
29 B	*S*. *aureus*	August 1, 2013	Surgery (laparotomy)	Wound swab	Surgical ICU	17	3
32	*S*. *aureus*	August 1, 2013	Bedsore	Wound swab	Surgical II	Corr	4
37 B	*S*. *aureus*	August 2, 2013	Injury-femur fracture	Wound Swab	Surgical II	8	3
38	*S*. *aureus*	August 2, 2013	Bedsores	Wound swab	Surgical II	8	2
56	*S*. *aureus*	August 13, 2013	Asthma + HIV	Blood	Medical I	2	4
59	*S*. *aureus*	August 15, 2013	Tumor wound	Wound swab	Surgical I	Corr	5
69 S	*S*. *aureus*	August 19, 2013	Burn wound	Wound swab	Surgical I	1	4
71	*S*. *aureus*	August 20, 2013	Abscess	Wound swab	Medical I	2	2
108	*S*. *aureus*	February 7, 2014	Pleural effusion (fever)	Blood	Medical II	11	3
143 A	*S*. *aureus*	March 11, 2014	Surgery (liver cirrhosis)	Wound swab	Medical I	5	6
153 C	*S*. *aureus*	March 17, 2014	Diabetes mellitus	Wound swab	Medical I	5	9
159 A	*S*. *aureus*	March 20, 2014	Abdominal tumor	Wound swab	Medical I	5	10
166	*S*. *aureus*	March 24, 2014	MTA	Wound swab	Surgical I	Corr	-
176 A	*S*. *aureus*	March 31, 2014	Diabetes mellitus	Wound swab	Medical I	5	Extra bed
196	*S*. *aureus*	April 11, 2014	Cellulitis	Wound swab	Surgical I	5	3
201 A	*S*. *aureus*	April 11, 2014	HIV (coughing)	Sputum	Medical I	2	Extra bed
204	*S*. *aureus*	April 15, 2014	Septicemia	Blood	Medical ICU	-	4
224 C	*S*. *aureus*	April 30, 2014	Diabetes mellitus	Wound swab	Medical II	Corr	6
260	*S*. *aureus*	June 2, 2014	Diabetes mellitus	Wound swab	Surgical I	5	8
309 B	*S*. *aureus*	September 19, 2014	Cellulitis	Wound swab	Surgical I	5	6
319 D	*S*. *aureus*	September 30, 2014	Burn	Wound swab	Surgical I	1	3
323 B	*S*. *aureus*	October 1, 2014	Diabetes mellitus	Wound swab	Surgical I	Corr	5
348 B	*S*. *aureus*	November 18, 2014	Tropical ulcer	Wound swab	Surgical I	5	6
349 A	*S*. *aureus*	November 19, 2014	Burn wound	Wound swab	Surgical I	1	1
437 C	*S*. *aureus*	March 10, 2015	Diabetes mellitus	Wound swab	Surgical I	5	7
449	*S*. *aureus*	March 31, 2015	Diabetes mellitus	Wound swab	Medical I	2	1
463 B	*S*. *aureus*	April 19, 2015	Surgery (laparotomy)	Wound swab	Surgical I	5	2
494	*S*. *aureus*	May 19, 2015	Burn wound	Wound swab	Surgical I	1	1
547	*S*. *epidermidis*	July 6, 2015	Septic wound	Wound swab	Surgical I	5	4
562	*S*. *haemolyticus*	July 15, 2015	HIV (cough)	Sputum	Medical I	2	1
564	*S*. *haemolyticus*	July 16, 2015	Diabetes mellitus	Wound swab	Surgical I	5	3
567 A	*S*. *aureus*	July 23, 2015	Septic wound	Wound swab	Surgical 1	Corr	5
577	*S*. *aureus*	August 3, 2015	Diabetes mellitus	Wound swab	Surgical I	2	Extra bed

**Table 2:** Phenotypic antimicrobial pattern of the 30 *S*. *aureus* isolates.

PATIENT ID	ORG	CTX	C	E	P	SXT	VA
WGS 029 B	*S*. *aureus *	R	S	R	R	R	S
WGS 032	*S*. *aureus*	R	S	R	R	R	S
WGS 037 B	*S*. *aureus*	S	S	S	R	S	S
WGS 038	*S*. *aureus*	S	S	I	R	S	S
WGS 056	*S*. *aureus*	R	S	R	R	R	S
WGS 059	*S*. *aureus*	S	S	R	R	I	S
WGS 069	*S*. *aureus*	R	S	R	R	R	S
WGS 071	*S*. *aureus*	R	S	R	R	R	S
WGS 108	*S*. *aureus*	R	S	R	R	R	S
WGS 143 A	*S*. *aureus*	R	S	S	R	S	S
WGS 153 C	*S*. *aureus*	R	S	S	R	R	S
WGS 159 A	*S*. *aureus*	S	S	S	R	S	S
WGS 166	*S*. *aureus*	S	S	S	R	S	S
WGS 176 A	*S*. *aureus*	S	S	S	R	S	S
WGS 196	*S*. *aureus*	S	S	R	R	R	S
WGS 201 A	*S*. *aureus*	S	S	I	R	S	S
WGS 204	*S*. *aureus*	S	I	I	R	S	S
WGS 224 C	*S*. *aureus*	S	S	S	R	S	S
WGS 260	*S*. *aureus*	S	S	S	R	S	S
WGS 309 B	*S*. *aureus*	R	S	R	R	R	S
WGS 319 D	*S*. *aureus*	R	S	R	R	R	S
WGS 323 A	*S*. *aureus*	S	S	R	R	S	S
WGS 348 B	*S*. *aureus*	R	S	R	R	R	S
WGS 349 A	*S*. *aureus*	R	S	S	R	R	S
WGS 437	*S*. *aureus*	S	S	S	R	S	S
WGS 449	*S*. *aureus*	S	S	S	R	S	S
WGS 463 B	*S*. *aureus*	S	S	S	R	R	S
WGS 494	*S*. *aureus*	R	S	R	R	R	S
WGS 567A	*S*. *aureus*	S	S	S	R	R	S
WGS 577	*S*. *aureus*	S	S	R	R	R	S
% resistance *n* (%)		13 (43.3)	0 (0)	14 (46.7)	30 (100)	16 (53.3)	0 (0)

C: Chloramphenicol, SXT: trimeth/sulfa, CXT: cefoxitin, E: erythromycin, P: penicillin G, VA: vancomycin, R: resistance, S: susceptible, and I: intermediate.

**Table 3:** Multilocus sequence typing, antimicrobial resistance genes, and virulence genes.

Sample name	Species	MLST	Resistance genes	Virulence factors
260	*S*. *aureus*	ST-1	*blaZ*	*splA, splB, splE, aur, hlb, lukE, lukD, seb, hlgB, hlgC, hlgA, sak, scn, se-h*
449	*S*. *aureus*	ST-1	*blaZ*	*splA, splB, splE, aur, hlb, lukE, lukD, seb, hlgB, hlgC, hlgA, sak, scn, se-h*
494	*S*. *aureus*	ST-1	*blaZ*	*splA, splB, splE, aur, hlb, lukE, lukD, seb, hlgB, hlgC, hlgA, sak, scn, se-h*
437 C	*S*. *aureus*	ST-1	*blaZ*	
577	*S*. *aureus*	ST-15	*aac(6*′*)-aph(2*′′*), ant(6)-Ia, aph(3*′*)-III, blaZ, dfrG, erm(B), erm(C), lsa(A)-like, tet(K), tet(M)*	*splA, splB, splE, aur, hlb, lukE, lukD, hlgB, hlgA, hlgC, scn.*
463 B	*S*. *aureus*	ST-15	*blaZ, dfrG, erm(C), tet(K)*	*splA, splB, splE, aur, hlb, lukE, lukD, hlgB, hlgA, hlgC, scn.*
196	*S*. *aureus*	ST-152	*blaZ-like, dfrG, erm(C), tet(K)*	*aur, hlb, hlgB, hlgA, sak, scan, lukF-PV, lukS-PV, edinB*
224 C	*S*. *aureus*	ST-152	*blaZ-like, dfrG, tet(K)*	*aur, hlb, hlgB, hlgA, sak, scan, lukF-PV, lukS-PV, edinB*
323 B	*S*. *aureus*	ST-152	*dfrG, erm(C), tet(K)*	*aur, hlb, hlgB, hlgA, sak, scan, lukF-PV, lukS-PV, edinB*
38	*S*. *aureus*	ST-1847	*blaZ-like*	*nil*
37 B	*S*. *aureus*	ST-1847	*blaZ*	*splA, splB, splE, aur, hlb, sek, seq, lukE, lukD, hlgB, hlgC, hlgA, sak, scn, lukF-PV, lukS-PV, sea/sep, sen, tst.*
176 A	*S*. *aureus*	ST-188	*blaZ-like, tet(K)-like*	*splA, splB, splE, aur, hlb, lukE, lukD, hlgB, hlgA, sak, scn.*
204	*S*. *aureus*	ST-22	*Not found*	*aur, hlb, hlgB, hlgA, hlgC, sak, scan, seo, sei, sen, seg, sem, tst, seu, *
201 A	*S*. *aureus*	ST-22	*Not found*	*aur, hlb, hlgB, hlgA, hlgC, sak, scan, seo, sei, sen, seg, sem, tst, enterotoxin, *
32	*S*. *aureus*	ST-239	*aac(6*′*)-aph(2*′′*)-like, ant(6)-Ia-like, aph(3*′*)-III, erm(A), mecA-like, spc-like, tet(M)*	Not found
29 B	*S*. *aureus*	ST-239	*aac(6*′*)-aph(2*′′*)-like, ant(6)-Ia-like, aph(3*′*)-III, erm(A), mecA-like, spc-like, tet(M)*	Not found
143 A	*S*. *aureus*	ST-30	*blaZ*	*splE, aur, hlb, hlgB, hlgC, hlgA, sak, scn, lukF-PV, lukS-PV, seo, sei, seu, sen, seg, sem*
567 A	*S*. *aureus*	ST-5	*blaZ, dfrG, tet(K)*	
166	*S*. *aureus*	ST-580	*blaZ*	*splA, splE, aur, hlb, lukE, lukD, hlgB, hlgA, hlgC, sak, scn.*
159 A	*S*. *aureus*	ST-6	*aadA2-like, aadB-like, blaCARB-2, blaZ, dfrA23, strA, strB, sul1, sul2, tet(31)-like, tet(G)*	
56	*S*. *aureus*	ST-8	*aac(6*′*)-aph(2*′′*), blaZ-like, dfrG, erm(C), mecA*	*splA, splB, splE, aur, hlb, sek, seq, lukE, lukD, seb, hlgB, hlgC, hlgA, sak, scn.*
71	*S*. *aureus*	ST-8	*aac(6*′*)-aph(2*′′*), blaZ, dfrG, erm(C), mecA*	*splA, splB, splE, aur, hlb, sek, seq, lukE, lukD, seb, hlgB, hlgC, hlgA, sak, scn.*
153 C	*S*. *aureus*	ST-8	*blaZ, dfrG, tet(K)*	*splA, splB, splE, aur, hlb, sek, seq, lukE, lukD, seb, hlgB, hlgC, hlgA, sak, scn, sea/sep.*
319 D	*S*. *aureus*	ST-8	*aac(6*′*)-aph(2*′′*), blaZ-like, dfrG, erm(C), mecA*	*splA, splB, splE, aur, hlb, sek, seq, lukE, lukD, seb, hlgB, hlgC, hlgA, sak, scn.*
348 B	*S*. *aureus*	ST-8	*aac(6*′*)-aph(2*′′*), blaZ-like, dfrG, erm(C), mecA*	*splA, splB, splE, aur, hlb, sek, seq, lukE, lukD, seb, hlgB, hlgC, hlgA, sak, scn.*
349 A	*S*. *aureus*	ST-8	*aac(6*′*)-aph(2*′′*), blaZ-like, dfrG, mecA*	*splA, splB, splE, aur, hlb, sek, seq, lukE, lukD, seb, hlgB, hlgC, hlgA, sak, scn.*
69 S	*S*. *aureus*	ST-8	*aac(6*′*)-aph(2*′′*), blaZ, dfrG, erm(C), mecA*	*splA, splB, splE, aur, hlb, sek, seq, lukE, lukD, seb, hlgB, hlgC, hlgA, sak, scn.*
59	*S*. *aureus*	ST-97	*blaZ-like, tet(K)*	*splA, splB, splE, aur, hlb, lukE, lukD, seb, hlgB, hlgC, hlgA, sak, scn.*
108	*S*. *aureus*	Unknown ST	*aac(6*′*)-aph(2*′′*), blaZ-like, dfrG, erm(C), mecA-like*	*splA, splB, splE, aur, hlb, sek, seq, lukE, lukD, seb, hlgB, hlgC, hlgA, sak, scn, sea/sep.*
309 B	*S*. *aureus*	Unknown ST	*aac(6*′*)-aph(2*′′*), blaZ-like, dfrG, erm(C), mecA-like*	*splA, splB, splE, aur, hlb, sek, seq, lukE, lukD, seb, hlgB, hlgC, hlgA, sak, scn, sea/sep.*
547	*S*. *epidermidis*	ST-59	*aac(6*′*)-aph(2*′′*), blaZ, erm(C), fosA, lnu(A)-like, mecA-like, tet(K)-like*	Not found
562	*S*. *haemolyticus*	ND	*aac(6*′*)-aph(2*′′*), blaZ-like, dfrG, erm(C), mecA, tet(K)-like, tet(M)*	Not found
564	*S*. *haemolyticus*	ND	*aac(6*′*)-aph(2*′′*), blaZ, dfrG, mecA-like, mph(C), msr(A)-like*	Not found

## Supplementary Materials

Supplementary 1 Figure 1a. BEAST tree using USA300_FPR37357 as a reference. Node labels indicate years.

Supplementary 2 Figure 1b. BEAST using USA500_2395 as a reference. Node labels indicate years.

Supplementary 3 Table 1a. Information of the six ST-8 MRSA genomes from Tanzania.

Supplementary 4 Table 1b. Information of the 34 ST-8 MRSA genomes from other parts of the world.
