# Supplementary figures and images for: Hospital Epidemiology of Methicillin-Resistant Staphylococcus aureus in a Tertiary Care Hospital in Moshi, Tanzania, as Determined by Whole Genome Sequencing
Source: Biomed Res Int. 2018 Jan 2;2018:2087693. doi: 10.1155/2018/2087693 (PMC5816877; doi:10.1155/2018/2087693)

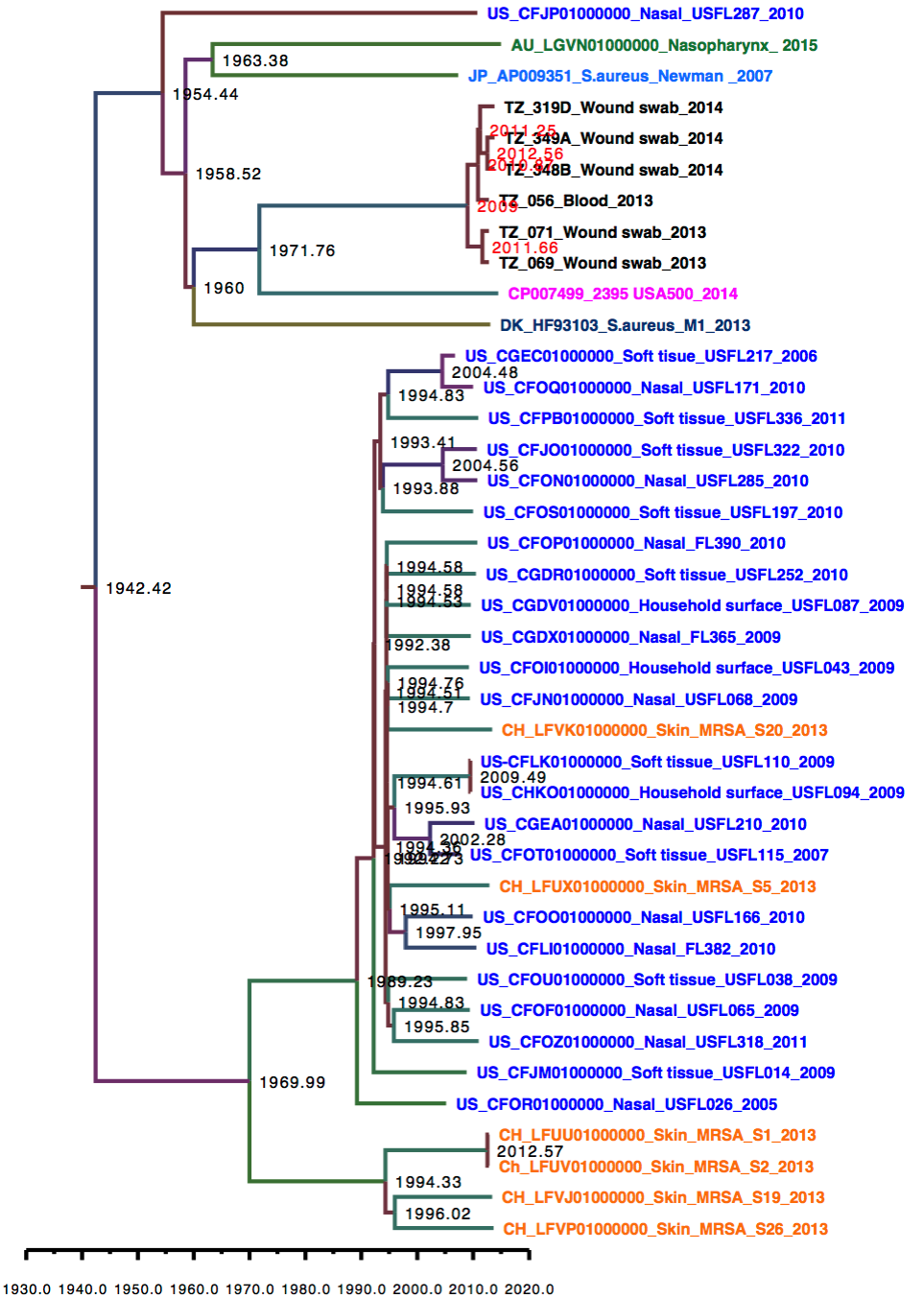


Figure 1a. BEAST tree using USA300_FPR37357 as a reference. Node labels indicate years

Supplement: Supplementary 1 — Figure 1a. BEAST tree using USA300_FPR37357 as a reference. Node labels indicate years. [file 2087693.f1.docx]

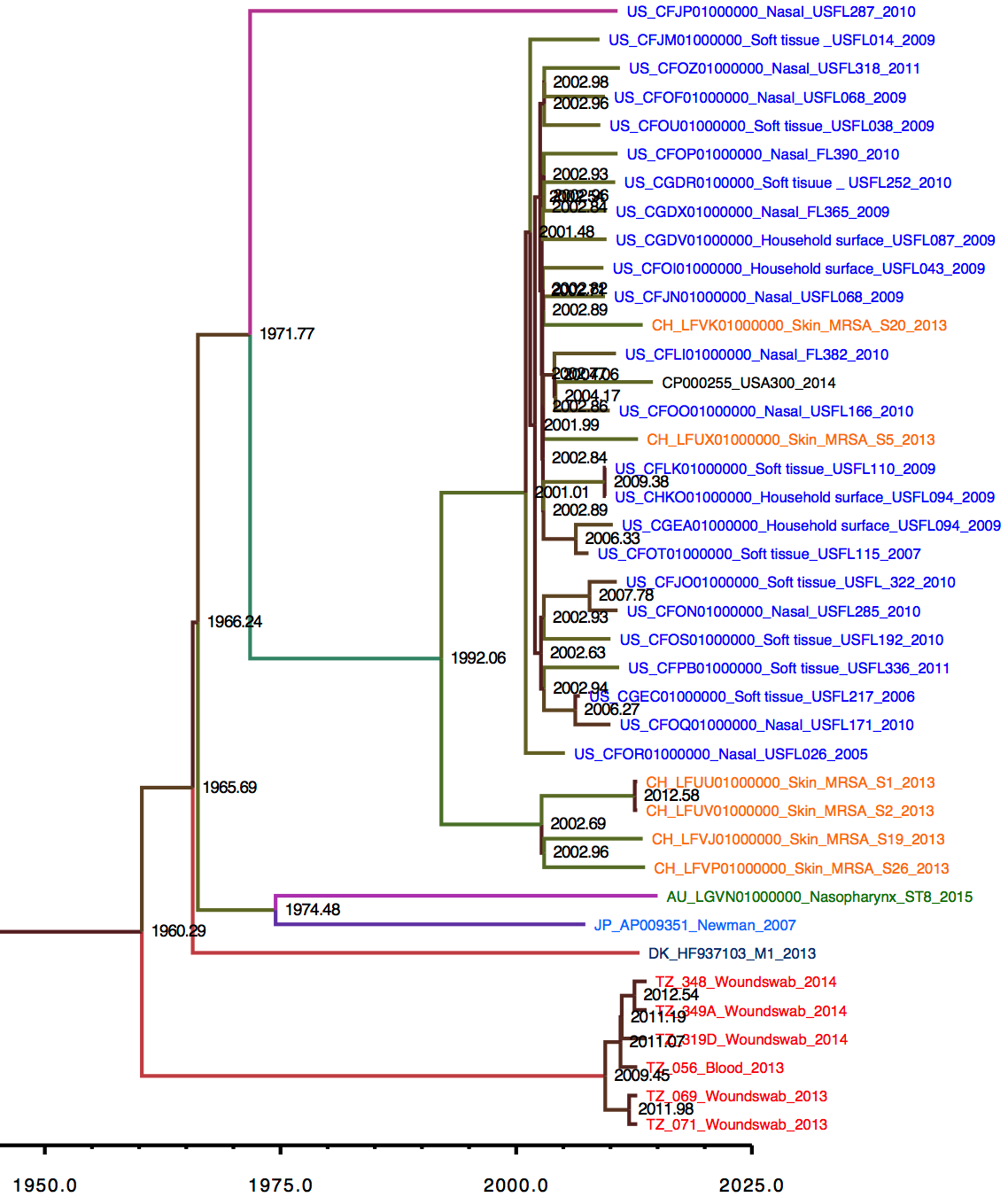


Figure 1b. BEAST using USA500_2395 as a reference. Node labels indicate years.

Supplement: Supplementary 2 — Figure 1b. BEAST using USA500_2395 as a reference. Node labels indicate years. [file 2087693.f2.docx]
