# Supplementary material for: Hospital Epidemiology of Methicillin-Resistant Staphylococcus aureus in a Tertiary Care Hospital in Moshi, Tanzania, as Determined by Whole Genome Sequencing
Source: Biomed Res Int. 2018 Jan 2;2018:2087693. doi: 10.1155/2018/2087693 (PMC5816877; doi:10.1155/2018/2087693)
Supplement: Supplementary 3 — Table 1a. Information of the six ST-8 MRSA genomes from Tanzania. [file 2087693.f3.docx]

Supplementary material

Table 1a: Information of the six ST-8 MRSA genomes from Tanzania

| **Strain** | **Species** | **Collection**  **Date** | **Host** | **Sequence**  **Type** | **Country** | **Source** | **Study Accession**  **Number** | **Sample**  **Accession**  **Number** |
| --- | --- | --- | --- | --- | --- | --- | --- | --- |
| TZWGS-56 | S.aureus | 13/8/13 | Human | ST-8 | Tanzania | Blood | PRJEB23314 | ERS1993316 |
| TZWGS-69S | S.aureus | 19/8/13 | Human | ST-8 | Tanzania | Wound swab | PRJEB23314 | ERS1993317 |
| TZWGS-71 | S.aureus | 20/8/13 | Human | ST-8 | Tanzania | Wound swab | PRJEB23314 | ERS1993318 |
| TZWGS-319D | S.aureus | 30/9/14 | Human | ST-8 | Tanzania | Wound swab | PRJEB23314 | ERS1993319 |
| TZWGS-348B | S.aureus | 18/11/14 | Human | ST-8 | Tanzania | Wound swab | PRJEB23314 | ERS1993320 |
| TZWGS-349A | S.aureus | 19/11/14 | Human | ST-8 | Tanzania | Wound swab | PRJEB23314 | ERS1993321 |
