# Supplementary material for: Hospital Epidemiology of Methicillin-Resistant Staphylococcus aureus in a Tertiary Care Hospital in Moshi, Tanzania, as Determined by Whole Genome Sequencing
Source: Biomed Res Int. 2018 Jan 2;2018:2087693. doi: 10.1155/2018/2087693 (PMC5816877; doi:10.1155/2018/2087693)
Supplement: Supplementary 4 — Table 1b. Information of the 34 ST-8 MRSA genomes from other parts of the world. [file 2087693.f4.docx]

Table 1b: Information of the 34 ST-8 MRSA genomes from other parts of the world

| Study no | Acc, number | Date of collection | Country | Source | Host | strain | ST | Spp |
| --- | --- | --- | --- | --- | --- | --- | --- | --- |
| PRJDA18801 | AP009351 | July,2007 | Tokyo, Japan | Genomic DNA | S.aureus | Newman | 8 | "Staphylococcus aureus" |
| PRJEB2870 | CFJM01000000 | January,2009 | USA:New York, Inwood | soft tissue | Human | USFL014 | 8 | "Staphylococcus aureus" |
| PRJEB2870 | CFOF01000000 | August,2009 | USA;New York, Inwood | nasal | Human | USFL068 | 8 | “Staphylococcus aureus” |
| PRJEB2870 | CFJN01000000 | November, 2010 | USA:New York, Inwood" | nasal | Human | USFL068" | 8 | "Staphylococcus aureus" |
| PRJEB2870 | CFJP01000000 | Dec, 2010 | USA:New York, Inwood" | nasal | Human | USFL287 | 8 | "Staphylococcus aureus" |
| PRJEB2870 | CFLI01000000 | OCTOBER,2010 | USA:New York, Inwood" | nasal | Human | FL382" | 8 | "Staphylococcus aureus" |
| PRJEB2870 | CFLK01000000 | Sept, 2009 | USA:New York, Inwood" | "soft tissue" | Human | "USFL110" | 8 | "Staphylococcus aureus" |
| PRJEB2870 | CFOF01000000 | August,2009 | USA:New York, CentralHarlem" | nasal | Human | "USFL065" | 8 | " Staphylococcus aureus" |
| PRJEB2870 | CFOI01000000 | June, 2009 | USA:New York, Inwood" | household surface" | Human | "USFL043 | 8 | "Staphylococcus aureus" |
| PRJEB2870 | CFON01000000 | Dec, 2010 | USA:New York, Inwood" | nasal | Human | "USFL285" | 8 | "Staphylococcus aureus" |
| PRJEB2870 | CFOO01000000 | Feb,2010 | USA:New York, CentralHarlem" | nasal | Human | "USFL166" | 8 | "Staphylococcus aureus" |
| PRJEB2870 | CFOP01000000 | Dec, 2010 | USA:New York, Inwood | nasal | Human | "FL390" | 8 | "Staphylococcus aureus" |
| PRJEB2870 | CFOQ01000000 | March,2010 | USA:New York, Inwood | nasal | Human | USFL171" | 8 | "Staphylococcus aureus" |
| PRJEB2870 | CFOR01000000 | May,2005 | USA:New York, Riverdale" | nasal | Human | "USFL026" | 8 | "Staphylococcus aureus" |
| PRJEB2870 | CFOS01000000 | March,2010 | USA:New York, CentralBronx" | "soft tissue" | Human | USFL197" | 8 | "Staphylococcus aureus" |
| PRJEB2870 | CFOT01000000 | Nov,2007 | USA:New York, Inwood" | "soft tissue" | Human | "USFL115" | 8 | "Staphylococcus aureus" |
| PRJEB2870 | CFOU01000000 | Feb,2009 | "USA:New York, Inwood" | "soft tissue" | Human | "USFL038" | 8 | "Staphylococcus aureus" |
| PRJEB2870 | CFOZ01000000 | March,2011 | USA:New York, Inwood" | nasal | Human | "USFL318" | 8 | "Staphylococcus aureus" |
| PRJEB2870 | CFPB01000000 | Feb,2011 | USA:New York, Inwood" | "soft tissue | Human | "USFL336" | 8 | "Staphylococcus aureus" |
| PRJEB2870 | CGDR0100000 | Sept, 2010 | "USA:New York, Inwood" | "soft tissue" | Human | USFL252" | 8 | "Staphylococcus aureus" |
| PRJEB2870 | CGDV01000000 | Sept, 2009 | "USA:New York, Morrisania" | household surface" | Human | USFL087" | 8 | "Staphylococcus aureus" |
| PRJEB2870 | CGDX01000000 | Sept, 2009 | USA:New York, Morrisania" | nasal" | Human | "FL365" | 8 | "Staphylococcus aureus" |
| PRJEB2870 | CGEA01000000 | June, 2010 | USA:New York, Inwood" | nasal | Human | "USFL210" | 8 | "Staphylococcus aureus" |
| PRJEB2870 | CGEC01000000 | Dec, 2006 | "USA:New York, Inwood" | "soft tissue" | Human | "USFL217" | 8 | "Staphylococcus aureus" |
| PRJEB2870 | CHKO01000000 | Sept, 2009 | USA:New York, Inwood" | "household surface" | Human | "USFL094" | 8 | "Staphylococcus aureus" |
| PRJNA240091 | CP007499 | Sept, 2014 | USA:New York, Inwood" | Genomic DNA | lab made | 2395 USA500" | 8 | "Staphylococcus aureus" |
| PRJNA288092 | LFUU01000000 | Jan,2013 | Switzerland:Geneva" | "skin" | Human | MRSA_S1" | 8 | "Staphylococcus aureus" |
| PRJNA288094 | LFUV01000000 | Jan,2013 | Switzerland:Geneva" | "skin" | Human | "MRSA_S2" | 8 | "Staphylococcus aureus" |
| PRJNA288096 | LFUX01000000 | Feb,2013 | "Switzerland:Geneva" | "skin" | Human | "MRSA_S5 | 8 | "Staphylococcus aureus" |
| PRJNA288110 | LFVJ01000000 | August,2013 | "Switzerland:Geneva" | skin | Human | "MRSA_S19" | 8 | "Staphylococcus aureus" |
| PRJNA288111 | LFVK01000000 | August,2013 | Switzerland:Geneva" | skin | Human | MRSA_S20" | 8 | "Staphylococcus aureus" |
| PRJNA288116 | LFVP01000000 | Nov,2013 | Switzerland:Geneva" | skin | Human | MRSA_S26 | 8 | "Staphylococcus aureus" |
| PRJNA291304 | LGVN01000000 | March,2015 | Australia:Brisbane" | nasopharynx sample | Human | "ST8" | 8 | "Staphylococcus aureus" |
| [PRJEB1620](https://www.ncbi.nlm.nih.gov/bioproject/PRJEB1620) | HF93103 | April,2013 | DENMARK | Genomic DNA, S.aureus | S.aureus | "M1" | 8 | "Staphylococcus aureus" |
